# Contributions to the History of the Small-Pox and of Vaccination in Denmark

**Published:** 1838-01

**Authors:** 


### Art. XXI.

*Bidrag til Bornekoppernes og Vaccinationens Historie i Danmark, og om de sidste herskende Koppe-Epidemier.* Af Joh. Chr. W. Wendt, m.d. —*Kiobenhavn,* 1836.

*Contributions to the History of the Small-pox and of Vaccination in Denmark; also Observations on the last prevailing Epidemics of Small-pox.* By J. C. Wendt, m.d.—*Copenhagen,* 1836. 8vo. pp. vi. 72.

The author of the above treatise had previously published an account of the small-pox and of the progress of vaccination in the Danish States. In the interval of the two publications, the author has acquired more experience,—has learned to estimate very highly the importance of vaccination,—and has acquired the conviction that the influence of time and of other circumstances is such as to diminish the protecting power of vaccination, so that it may require to be repeated more than once. Epidemics of small-pox have, during this interval, been prevalent; a system of quarantine, and a mode of treatment corresponding to it, have been adopted, and again given up. As the author (between May 15, 1835, and March 31, 1836,) in his capacity of superintendent physician to the hospitals of Copenhagen, was engaged in the treatment of 1389 cases of variola, his opportunities for collecting materials worthy of communication on the subject of this treatise must have been considerable. In the epidemic of variola which commenced in March, 1828, and which prevailed, with a short interval, until July, 1830, 562 patients were received and treated in the hospital; of whom, 111 had the true variola. Of these there died 29,—in the proportion therefore of 1 in 4: 84 had not been vaccinated, and of these 24 died,—in the proportion of 1 in 3½. One of them did not know if he had been vaccinated; and 29 were specified as vaccinated, of whom only 4 died,—i. e. 1 in 7. This confirms the fact that the previous vaccination, even if it had not produced any modification in the outward form of the disease, had materially influenced its mortality.

Among the patients who had been vaccinated, there was only one of four and a half years of age: all the others were older, *and most of them had been vaccinated in the early period, when vaccination was extensively employed.* The younger the patients were, so much the more mild was the variola. None of the vaccinated children died, or had true small-pox.

In August, 1832, commenced the most violent epidemic of small-pox which has prevailed in Copenhagen since the introduction of vaccination. Until the end of the year 1834, as many as 1045 patients were received into the small-pox hospital, of whom 45 died; among them was a female, who died nineteen days after the eruption of the pock, and sixteen days subsequent to her delivery. The proportion of deaths was 1 in 24. The number of individuals not vaccinated, and of those who were vaccinated eight days previous to the breaking out of the small-pox was 147; of whom 34 died,—i. e. 1 in 4. Eight hundred and ninety-eight had been vaccinated, and of these 10 only died,—i. e. 1 in 90. Of 179 patients who had the true disease, 119 had not been vaccinated; of these there died 34, or 1 in 3½; 60 had been vaccinated, and 10 of them died, or 1 in 6; which confirms the protecting power of vaccination. In both epidemics, the efficacy of vaccination was seen; but experience proved that in many cases the protecting power was weakened after many years; and this was shown by the modification of the pock in infants and adults; for the disease was only seen in its true form in such as had been vaccinated and had completed their fourteenth year, and of those who had been vaccinated none died of small-pox before they were twenty-three years of age. Again, the pock was often so far modified that it never burst; and, of those who were revaccinated, not one had the disease.

On the 1st of May, 1835, the quarantine against the disease was terminated, although the disease itself continued to advance. In one hospital, between the 15th May and the end of the year 1835, the author treated—vaccinated patients, 1043; non-vaccinated, 123. In addition to these were 31, in whom no traces of vaccination or of true pock could be discovered, making together a total of 1197. Although the pustules of those patients who had been vaccinated were generally of the milder kind (varioloid), still there were occasionally cases which almost completely exhibited the true matured pustule, and even confluent in a high degree. Of the whole number of the 1043 vaccinated, there died 47; three of whom were in their eighteenth and nineteenth years: none were younger than this. Of the 123 who were not vaccinated, partly because they had had the small-pox, 51 died; i. e. five children beneath a year old; two within ten years of age; the other 44 between their twenty-fifth and fifty-second years. Of the 31 cases of small-pox in which previous vaccination or small-pox was quite uncertain, eight died; none being less than ten years of age, nor beyond forty-three, excepting one individual, aged fifty-three. Of the vaccinated there died about one in 22; whilst, of those who had not been vaccinated, about one-third fell a sacrifice to the disease. It must also be remarked, that the small-pox patients received into the hospital of which we have just spoken, had usually, at the period of reception, reached an advanced stage of the disease; some of them having lain days, or even weeks, in their miserable abodes, and others being even at the point of death. If those who were dying when brought to the hospital, together with those who lost their lives by morbid complications of the small-pox, are subtracted, it would leave the proportion of 58 deaths in 1149 patients who were treated, which must be regarded as a comparatively small mortality.

Revaccination is introduced into the Danish army, as an effectual means of diminishing the contagion of variola, and of securing against the fatal consequences of the disease. The result in the year 1835 was as follows:

| Age. | Revaccinated with effect. | Revaccinated without effect. |
|---|---|---|
| 1—10 years. | 33 individuals. | 1 individual. |
| 10—20 | 216 | 82 |
| 20—25 | 2175 | 998 |
| 25—30 | 191 | 76 |
| 30—40 | 123 | 43 |
| 40—50 | 18 | 8 |

It is very important, both in vaccination and revaccination, that the operation should be performed with great exactness, and with lymph transmitted through healthy subjects. Winslow received the first lymph from London, on July 6, 1801; and since that time it has been transplanted from individual to individual, and, as may be learned from the experiments and their results during the long epidemics of small-pox, it has continued unweakened in its protecting power. In respect to that question, so often asked, Whether it is fitting to take the matter originally from the cow? the opinion of the author is, that (since the erroneous notion that vaccination protected during the whole life has been given up,) the circumstances have in so far become changed, that, from the acknowledged advantages derived from vaccination, there is so great a demand for the matter, that it must be necessary in some instances to obtain it from the cow itself. He reports that Dr. Ritter, the superintendent of the institution for vaccination in Kiel, in consequence of a royal command, had undertaken several journeys to those places in Holstein where the cows had the pock; and that he had sent to the Sanatory College in Copenhagen some of the vaccine matter which he had taken from the cow, which succeeded well. Dr. Ritter has remarked that the vaccinations which he performed with the matter of the cow-pock, in the years 1824, 1826, 1829, 1830, and 1832, upon children, produced larger pocks than those produced in the ordinary manner; and that they were surrounded by a strongly inflamed areola. The cicatrices, also, which remained after these, were more marked and deeper than in the common form. Herhold says, in a letter of the 6th of December, 1834, addressed to the author, "that he entertains no doubt that vaccination from young cows might be the most appropriate method, both to renew the vaccine matter and to provide the requisite quantity of it for revaccination."

From his collected experience the author draws the following conclusions: That the true or inoculated small-pox, which has run through its course, does not certainly protect; that the same individual (though such a case is rare,) may be a second time affected with the disease; that such is more frequently the case after vaccination, although the disease is then usually the varioloid; that the protecting power of a good vaccination generally secures for a certain time, perhaps for six years, and that a person who has been properly vaccinated always has a milder form of the disease; that, on those accounts, revaccination should be performed. The time at which the protecting power of the vaccine terminates cannot be correctly estimated: it depends on the susceptibility of the system of each individual for the small-pox. The revaccination must therefore be undertaken when an epidemic of small-pox prevails; and, should not the vaccination answer at this time, it must be repeated at a longer or shorter interval. It is also certain that a fatal termination of the disease is rare when the vaccination and revaccination have been perfectly performed and no causes tend to prevent its favorable operation; and, consequently, that the discovery of Jenner is of great advantage to mankind.

The very general prevalence of epidemic small-pox throughout England of late years, and the now generally acknowledged inefficacy of vaccination to guard against it in a large proportion of cases, renders every contribution to our knowledge, on this subject, of importance in the present state of the enquiry. We have on this account deviated, on the present occasion, from our invariable custom of writing our notices of books from the original. Dr. Wendt's book not having reached us, we have translated the above brief analysis of it, by Dr. Von Schönberg, of Copenhagen, from *Smidt's Jahrbucher*, which we give without comment. On the same grounds of utility, we shall give here, rather than in our department of Selections, the following Report from a late Number of the *Medicinische Zeitung*.

*Results of Revaccination of the Prussian Army in* 1836. By Dr. Schlesier.

"In this year, 42,124 individuals were revaccinated. Of this number, former cicatrices were distinct in 32,635, indistinct in 6,645, and none were apparent in 2,844. In the revaccinations of this year, regular pustules were produced in 18,136 cases, irregular in 9,940, and in 14,048 the inoculation was at first unsuccessful, but, on repetition, succeeded in 1,569, and remained abortive in 8,205 cases. The number of pustules on each individual varied from one to thirty; of those on whom regular pustules were produced, 7,311 had from one to five pustules; 5,647 from six to ten; 4,418 from eleven to twenty; and 760 from twenty-one to thirty.

"Of cases revaccinated in 1836, or at an earlier period, 14 had during this year an attack of varicella, and 8 of the varioloid disease; but there was no case of genuine small-pox.

"The inoculation was in general performed with fresh matter taken from the arms of infants; sometimes it was necessary to draw the matter from the arm of adults, and only very seldom was it necessary to have recourse to preserved matter. The surgeons of different corps differed in opinion as to the superior or inferior efficacy of matter drawn from the infant or adult, but direct experiment seemed to shew very little difference in their respective powers of producing pustules.

"Compared with former years, the results of the revaccination of 1836 seem to lead to the conclusion that the susceptibility to the vaccine disease, and consequently also to small-pox, is becoming annually greater. Of 42,124 individuals revaccinated in 1836, genuine pustules were produced in 18,136; in 1833, 48,478 individuals were revaccinated, and of these 15,269 with success; in 1834, of 44,454 cases, 16,679 were revaccinated successfully; and, in 1835, of 39,192 cases, success was obtained in 15,315. Or, of 100 revaccinations of each year, 31 were successful in 1833, 37 in 1834, 39 in 1835, and 43 in 1836.

"In 1833, 4,161 were revaccinated unsuccessfully, but a repetition of the inoculation was successful in 784; of 4,830 unsuccessful cases in 1834, the repetition was successful in 866; in 1835, in 9,411 unsuccessful cases, it succeeded in 1465; and 1836, in 1,569 out of 9,744." *Medicinische Zeitung*, 1837. No. 20 and 21.

The results of the revaccination of 1834 are given in our Third Number.

